# Bone mineral density assessment using iterative reconstruction compared with quantitative computed tomography as the standard of reference

**DOI:** 10.1038/s41598-018-33444-5

**Published:** 2018-10-10

**Authors:** Constanze Mann, Katharina Ziegeler, Jürgen Mews, Martina Plaschke, Ahi Sema Issever

**Affiliations:** 1 Department of Radiology, Charité – Universitätsmedizin Berlin, Berlin, Germany; 2 Canon Medical Systems Europe BV, Zoetermeer, Netherlands; 3 Department of Anatomy, Charité - Universitätsmedizin Berlin, Berlin, Germany

## Abstract

This study examines the influence of iterative reconstruction on bone mineral density (BMD) measurement by comparison with standard quantitative computed tomography (QCT; reference) and two other protocols based on filtered back projection. Ten human cadaver specimens of the lumbar spine with a hydroxyapatite calibration phantom underneath, were scanned with 4 protocols: 1. standard QCT, 2. volume scan with FBP, 3. helical scan with FBP, and 4. helical scan with IR (Adaptive Iterative Dose Reduction 3D (AIDR3D)). Radiation doses were recorded as CT dose index (CTDIvol) and BMD, signal-to-noise and contrast-to-noise ratio were calculated. Mean hydroxyapatite concentration (HOA) did not differ significantly between protocols, ranging from 98.58 ± 31.09 mg cm^3^ (protocol 4) to 100.47 ± 30.82 mg cm^3^ (protocol 2). Paired sample correlations of HOA values for protocol 4 and protocols 1, 2 and 3 were nearly perfect with coefficients of 0.980, 0.979 and 0.982, respectively (p < 0.004). CTDIvol were 7.50, 5.00, 6.82 (±2.03) and 1.72 (±0.50) mGy for protocols 1, 2, 3 and 4 respectively. Objective image quality was highest for protocol 4. The use of IR for BMD assessment significantly lowers radiation exposure compared to standard QCT and protocols with FBP while not degrading BMD measurement.

## Introduction

Osteoporosis is a systemic skeletal disease characterized by a low bone mineral density (BMD) and an associated increase in the risk of fracture^1^. Since the prevalence of osteoporosis is increased with the aging population and osteoporotic fractures have a high morbidity and mortality, diagnosis of osteoporosis at an early stage becomes more and more important^2,3^.

It has been shown that BMD (milligrams per cubic centimeter) is a good predictor of fracture risk^4,5^. As bone loss first appears in trabecular bone with high turnover rates such as the vertebral body, the main concern in spinal bone BMD assessment is the vertebral body^6^.

The only reference method accepted by the WHO to measure BMD is dual energy X-ray absorptiometry (DXA), a two-dimensional method which indirectly quantifies calcium from specific absorption values. DXA measures BMD by projection, thus also including cortical bone, spondylophytes or even aortic calcifications, which can lead to false negative results and is one of the main reasons why osteoporosis is underdiagnosed^7–9^.

A well-established alternative to DXA is quantitative computed tomography (QCT), a three-dimensional method which measures trabecular BMD in milligrams per cubic centimeter by indirectly quantifying hydroxyapatite in comparison to a reference phantom. Cortical bone, spondylophytes and aortic calcifications are not included in the measurement due to its three-dimensional character. In addition, QCT provides more information on geometric and structural parameters of bone, which contribute to skeletal strength. Nevertheless, a limitation of QCT is the relatively high radiation dose associated with scanning central body sites (spine and hip) compared to DXA, but doses are comparable to other x-ray-based imaging methods that might be performed in this patient group (e.g., spinal radiographs)^10^.

Several studies evaluted the use of routine thoracic or abdominal CT scans for assessing BMD^11–15^, but opinions are divided regarding the effect of contrast media on bone density measurement^13,14^.

In the last few years, considerable advances have been made in CT with iterative reconstruction (IR). Compared to filtered back projection (FBP), which for a long time was the only image reconstruction technique for CT but had the disadvantage of generating relatively noisy images, iterative reconstruction techniques substantially lower image noise and this can be exploited to improve image quality or to lower radiation dose^16–19^. Recent studies validated the use of IR in different imaging applications^20–23^.

Some studies investigated the impact of IR in musculoskeletal CT^24–28^ and a few studies investigated the impact of IR on trabecular bone microstructure assessment^29–31^.

We conducted a study to compare BMD determined from iteratively reconstructed CT datasets with BMD calculated from QCT - the standard reference method.

## Methods

### Study Design

Ten human cadaver specimens of the lumbar spine without surrounding soft tissue fixed in 4% paraformaldehyde were obtained from body donors (five women and five men; mean age 80 years, range 59–92 years). The specimens were obtained from the Department of Anatomy, Charité Campus Mitte, Universitätsmedizin Berlin, Germany.

According to the standard procedure of bone density measurement in QCT, lumbar vertebrae 1–3 were evaluated. Lumbar vertebra 1 of one specimen needed to be excluded due to fracture. Therefore, a total of 29 lumbar vertebrae were evaluated.

### Scanning Technique and Image Reconstruction

All examinations were performed on a 320-row CT scanner (Aquilion ONE Vision^TM^, Canon Medical Systems, Otawara, Japan). All specimens were fixed and scanned in a water bath. Underneath the water bath a hydroxyapatite (HA) calibration phantom consisting of five tubular inserts with varying concentrations of HA (0, 50, 100, 150 and 200 mg/cm^3^) was placed. The vertebrae were scanned and reconstructed parallel to the upper and lower plates of the vertebrae. The vendor unique IR is called Adaptive Iterative Dose Reduction 3D (AIDR3D), we used the level standard for reconstruction. This IR algorithm is a hybrid model algorithm, a full model based reconstruction algorithm was not available at the time we performed the scan and reconstruction. Four different scan protocols were used. Protocol 1 (QCT; sequential scan) serves as the reference in this study and our institute since more than 15 years. Here only the BMD value is of interest no volume information from the vertebrae i.e. for fracture analysis. Following parameter: 80 kVp, 200 mAs, acquired 4 × 2 mm, reconstructed in 8 mm FBP with a soft tissue kernel (FC13) without beam hardening correction. From Protocol 2 to 4 we have acquired the full vertebrae and reconstructed the same slice location given by the sequential scan. Protocol 2 (volume scan, single gantry rotation): 80 kVp, fixed mA, acquired 120 × 0.5 mm, reconstructed in 8 mm with FC13. Protocol 3 (helical scan): 80 kVp, automatic exposure control based on FBP reconstruction with an image noise level of 20, acquired 64 × 0.5 mm and Pitch of 0.83, reconstructed in 8 mm with FC13. Protocol 4 (helical scan): 80 kVp, automatic exposure control based on AIDR3D standard reconstruction with an image noise level of 20, acquired 64 × 0.5 mm and Pitch of 0.83, reconstructed in 8 mm with FC13. The specific scan parameters are listed in Table 1. Radiation doses were recorded as CT dose index (CTDIvol) in mGy as this is an adequate parameter for comparison against the reference protocol, which is scanned in sequential scan technique. Volume acquisition information, which is mandatory for assessing structural parameter, is not part of this paper, we focusing on one single plane only.

**Table 1:** Acquisition technique and scan parameters of the four CT scan protocols used in this study.

Protocol	Acquisition technique	kVp	Time [s]* Current [mA]	Reconstruction algorithm	Covered axial thickness (mm)	Image thickness (mm)	CTDI_vol_ [mGy]
1 (QCT)	Axial	80	200	FBP	8	8	7.5
2	Volume	80	200	FBP	60	8	5.0
3	Helical	80	Variable-AEC	FBP	64	8	6,8
4	Helical	80	Variable- AEC	iterative	64	8	1,7

Protocol 1 (QCT) served as reference. AEC (Automatic Exposure Control) adapts the tube current to the body shape and tissue absorption. The modulation ranges from 10 mA to 800 mA.

### Image Evaluation

**Figure 1:**
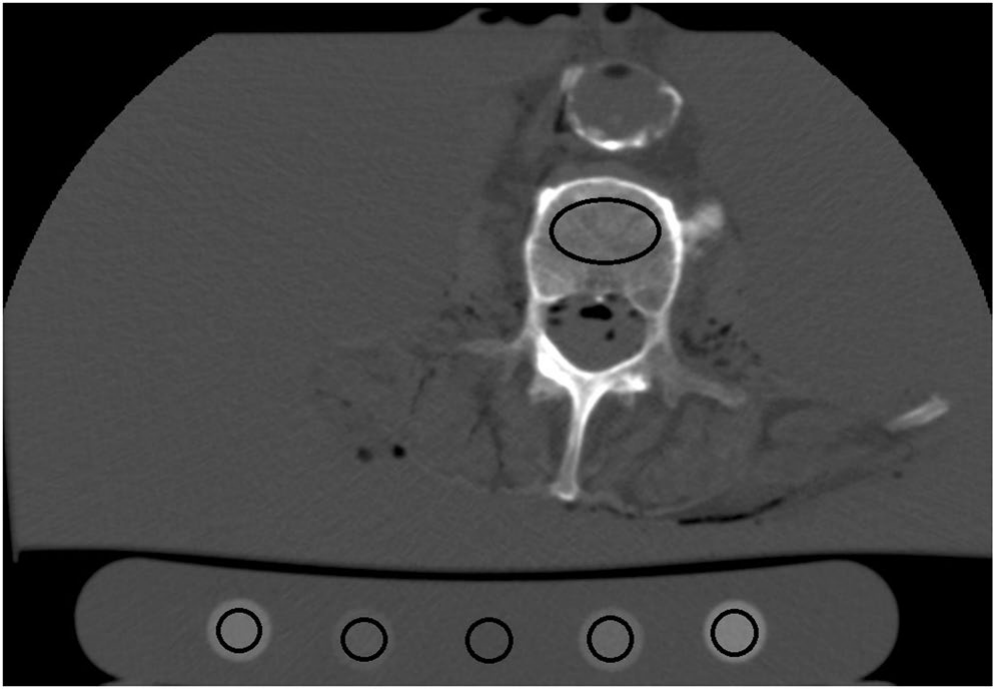
Image evaluation and analysis: Circular regions of interests (ROIs; 5 mm diameter) were drawn in each hydroxyapatite cylinder and a bigger oval ROI in the anterior 2/3 of the vertebra.

One radiologist reviewed all image datasets. The reviewer was a resident with 2.5 years of experience. No time limits were set for image review. Measurements were performed at an advanced workstation from Canon Medical Systems, Software Version V 6 SP 0010G. Circular regions of interests (ROIs; 5 mm diameter) were drawn in each hydroxyapatite cylinder and a larger oval ROI in the anterior 2/3 of the vertebra. The ROIs in the vertebrae were drawn to be as large as possible, carefully avoiding compact bone and the venous plexus of the vertebra. The size and position of the ROIs were kept constant in each measurement in a vertebra. An example for illustration is shown in Fig. 1.

### Bone Mineral Density and Markers of Objective Image Quality

Mean attenuation (in Hounsfield units = HU) and standard deviation (SD) were recorded for each vertebra and the five phantom ROIs. Applying linear regression analysis BMD was calculated as the hydroxyapatite concentration (HOA) for each vertebra in each scan and reconstruction mode.

For objective assessment of image quality, signal-to-noise ratio (SNR) and contrast-to-noise ratio (CNR) were calculated as follows:

$$\begin{array}{rcl}{\rm{Image}}\,{\rm{noise}}\,(\mathrm{IN}) & = & {\rm{SD}}\,{\rm{of}}\,{\rm{HU}}\,0\,{\rm{mg}}/{{\rm{cm}}}^{3}\,{\rm{HA}}\,{\rm{phantom}}\\ {\rm{SNR}} & = & {\rm{mean}}\,{\rm{vertebral}}\,{\rm{HU}}/{\rm{IN}}\\ {\rm{CNR}} & = & ({\rm{HU}}\,{{\rm{Mean}}}_{{\rm{bone}}}-{\rm{HU}}\,{{\rm{Mean}}}_{{\rm{phantom}}{\rm{HOA}}50})/{\rm{IN}}\end{array}$$

### Statistical Analysis

Means and standard deviations for HOA and CTDIvol as well as IN, SNR and CNR were calculated for all protocols. Differences in means between protocol 4 and all other protocols were tested for significance, using Student’s t-test for paired samples, including calculation of paired sample correlation coefficients. To account for multiple comparisons (n = 12), Bonferroni correction was applied. Therefore, the significance level assumed for all tests was p < 0.004. All analyses were performed using SPSS Version 22 (IBM Corporation, New York, USA).

The datasets generated and analysed during the current study are available from the corresponding author on reasonable request.

The entire study design and conduct were approved by the Charité ethics committee (EA1/031/17). Before death, all donors had given written consent to dedicate their bodies to research. All methods were carried out in accordance with relevant guidelines and regulations.

## Results

### Bone Mineral Density and Radiation Dose

**Figure 2:**
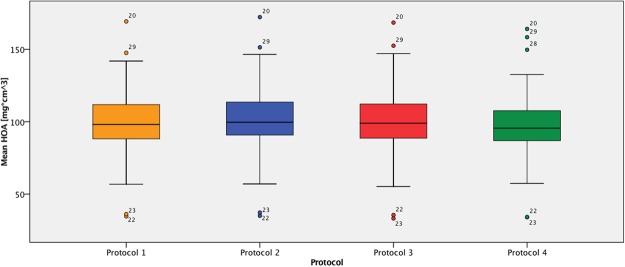
Mean HOA [mg cm^3^]. Boxplot with quartiles. Protocol 1 (=orange), protocol 2 (=blue), protocol 3 (=red), protocol 4 (=green). Outliers labeled by vertebral ID.

**Figure 3:**
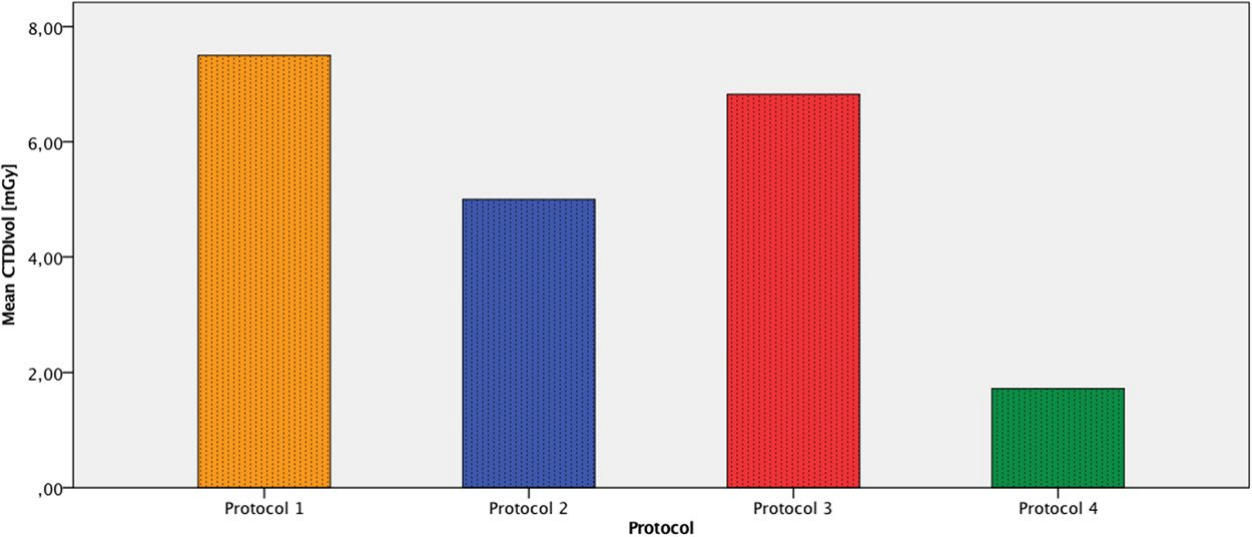
Mean CTDI_vol_ [mGy]. Bar charts. Protocol 1 (=orange), protocol 2 (=blue), protocol 3 (=red), protocol 4 (=green). Outliers labeled by vertebral ID.

Mean HOA did not differ significantly between protocols, ranging from 98.58 ± 31.09 mg cm^3^ (protocol 4) to 100.47 ± 30.82 mg cm^3^ (protocol 2). Paired sample correlations of HOA values for protocol 4 versus protocols 1, 2 and 3 were nearly perfect with coefficients of 0.980, 0.979 and 0.982, respectively (p < 0.004). A graphical representation of mean HOA values is presented in Fig. 2. Radiation doses expressed as CTDIvol were 7.50, 5.00, 6.82 (±2.03) and 1.72 (±0.50) mGy for protocols 1, 2, 3 and 4, respectively. The difference between protocol 4 and the other protocols was statistically significant (p < 0.004). A graphical representation of these results is presented in Fig. 3.

### Markers of Objective Image Quality

Mean values of markers of objective image quality are compiled in Table 2. The results show that image quality was significantly higher for protocol 4 compared with QCT and protocols with filtered back projection.

**Table 2:** Image quality. SNR = signal-to-noise ratio; CNR = contrast-to-noise ratio.

Protocol	Image noise	SNR bone	CNR bone
Protocol 1	16.40 ± 3.82	9.01 ± 3.64	5.28 ± 3.68
Protocol 2	13.22 ± 3.16	11.18 ± 4.55	6.76 ± 4.54
Protocol 3	13.27 ± 1.97	11.01 ± 3.86	6.44 ± 4.20
Protocol 4	8.48 ± 1.47	17.99* ± 7.46	10.39* ± 6.97

Means with standard deviations. *Significantly higher (p < 0.004) than in the other protocols.

## Discussion

The aim of our study was to evaluate the effect of IR on bone mineral density assessment and its different scan mode acquisition in comparison to standard (sequential) QCT and protocols reconstructed with FBP. Our results show that the use of IR for bone mineral density assessment significantly increases objective image quality and significantly decreases radiation dose compared to standard QCT and protocols with FBP while, most importantly in the context of our current study, the measured BMD remains the same. This goes in line with a recent study of Mei *et al*. who analyzed the effect of lower tube currents and sparse samplings on BMD and quantitative bone microstructure assessment *in-vivo*. They showed that there was no significant change in BMD when analyzing the reduced projections even at 10% sampling rate, whereas lowering tube current to 10% resulted in an average 38% higher BMD values, suggesting sparse sampling to be a more robust dose reduction approach for BMD measurements^29^. On the other hand, Anitha *et al*. showed that radiation dose (by tube exposure reduction) could be reduced to 64% with no impact on strength estimates obtained from finite-element- (FE-) analysis and no significant difference in volume quantification^32^. Evidence of the dose reduction potential of IR is also provided by other studies of musculoskeletal CT: Geravaise *et al*. demonstrated that the radiation dose for lumbar spine CT imaging could be reduced by 52%^33^. In a retrospective CT study of the cervical spine using FBP and IR, Geyer *et al*. found that the estimated mean effective dose decreased from 2.38 mSv (FBP) to 1.10 mSv (IR), concluding that radiation dose can be reduced to the level of plain radiography^27^. In our cohort we could reduce the radiation dose by 77% without a loss of accuracy in BMD measurement.

Other studies focus on dose reduction with iterative reconstruction for the assessment of trabecular bone microstructure in multi-detector CT (MDCT)^29–31^. The high diagnostic value of microstructure-analysis in MDCT is limited by high radiation doses of about 3 mSv^34^. Kopp *et al*. showed that trabecular bone microstructure parameters assessed by low-dose IR significantly correlate with vertebral bone strength and were not significantly different to parameters assessed by standard-dose FBP in MDCT^30^. Mookiah *et al*. found that different exposures (80, 150, 220 and 500 mAs, 100% projections) between FBP and IR produce similar texture values and quality, and on the other hand, images reconstructed from sparse sampling projections (50%, 25%, 12,5%), standard (220 mAs, 100% projections) and reduced (150 mAs, 100% projections and 80 mAs, 100% projections) exposures using IR produce a texturally comparable image quality^31^.

In our study, objective IN was significantly lower and SNR and CNR significantly higher for protocol 4 (reconstructed by IR) compared to the other three protocols (reconstructed by FBP). These results agree well with published data: in a prospective study of 34 patients scanned with unenhanced CT (reconstructed with FBP, hybrid IR, and model-based iterative reconstruction (MBIR)) and magnetic resonance (MR) imaging for lumbar canal spinal stenosis, Iyama *et al*. found a significant reduction of image noise and a significant increase of CNR for MBIR compared to FBP and IR^24^. In a prospective study of 40 patients undergoing cervical spine CT and randomized to a standard dose (SD, 120 kVp, 275 mAs) and a reduced dose group (RD, 120 kVp, 150 mAs), Becce at al. showed that IR significantly decreased image noise and increased SNR and CNR compared with FBP. Moreover, SNR and CNR were statistically equivalent in RD-IR and SD-FBP^26^.

IR could not be performed in sequential QCT at the time of acquisition and evaluation, which could have been an interesting comparison. In current software releases for the CT-Scanner this would be possible.

Not in the scope of our paper is the axial coverage, which is of course also important for obtaining further diagnostic information, such as structural analysis information and/or finite element analysis, where a fracture threshold can be determined. For this kind of analysis a single rotation of a volume or a helical acquisition is necessary to cover the whole vertebra, as a sequential scan is not sufficient. This should also be noted when comparing the radiation dose between protocols 1 and 4. Further radiation dose reduction is possible by reducing the axial scan range of protocol 2 to 4.

Another limitation of our study is the small sample size, which is due to the study design (only 10 human cadaver specimens of the lumbar spine could be provided by the anatomical institute during the study period). Second, our study can only serve as a model because the measurements made do not reflect true BMD measurement in patients due to the absence of surrounding tissues in the specimens. In addition, due to the setup in the waterbath scattering is very homogeneous and thus artifacts that may compromise the quantification at low dose levels might be smaller than in an *in-vivo* setting.

During the time of acquisition and reconstruction of the study the latest development of iterative reconstruction, a forward projected model based iterative reconstruction solution (FIRST) was not available at site so we cannot judge on this.

Nevertheless, valid conclusions can be drawn and serve as basis for further studies – eventually in patients: bone mineral density remains the same in QCT, CT datasets reconstructed with FBP and CT datasets reconstructed with IR, while radiation dose can be reduced significantly by IR independent of the acquisition mode.

## References

[1] World Health Organization (2003). Prevention and management of osteoporosis: report of a WHO scientific group. Worls Health Organ Tech Rep Ser..

[2] Cole ZA, Dennison EM, Cooper C (2008). Osteoporosis epidemiology update. Curr Rheumatol Rep..

[3] Leboime A (2010). Osteoporosis and mortality. Joint Bone Spine..

[4] World Health Organization. WHO scientific group on the assessement of osteoporosis at primary health care level. World Health Organization, 1–17 (2007).

[5] Ross PD, Davis JW, Vogel JM, Wasnich RD (1990). A critical review of bone mass and the risk of fractures in osteoporosis. Calcif Tissue Int..

[6] Cummings SR, Bates D, Black DM (2002). Clinical use of bone densitometry: scientific review. JAMA..

[7] Antonacci MD, Hanson DS, Heggeness MH (1996). Pitfalls in the measurement of bone mineral density by dual energy x-ray absorptiometry. Spine..

[8] Wang Y, Videman T, Boyd SK, Battie MC (2015). The distribution of bone mass in the lumbar vertebrae: are we measuring the right target?. Spine J..

[9] Chesnut CH (2001). Osteoporosis, an underdiagnosed disease. JAMA..

[10] Adams JE (2009). Quatintitative computed tomography. Eur J Radiol..

[11] Marinova M (2015). Use of routine thoracic and abdominal computed tomography scans for assessing bone mineral density and detecting osteoporosis. Curr Med Res Opin..

[12] Lee SJ (2016). Opportunistic screening for osteoporosis using the sagittal reconstruction from routine abdominal CT for combined assessment of vertebral fractures and density. Osteoporos Int..

[13] Pickhardt PJ (2016). Effect of IV contrast on lumbar trabecular attenuation at routine abdominal CT: correlation with DXA and implications for opportunistic osteoporosis screening. Osteoporos Int..

[14] Pompe E (2015). Intravenous contrast injection significantly affects bone mineral density measured on CT. Eur Radiol..

[15] Pickhardt PJ (2013). Opportunistic screening for osteoporosis using abdominal computed tomography scans obtained for other indications. Ann Intern Med..

[16] Leipsic J (2010). Estimated radiation dose reduction using adaptive statistical iterative reconstruction in coronary CT angiography: The ERASIR study. AJR Am J Roentgenol..

[17] Moscariello A (2011). Coronary CT angiography: image quality, diagnostic accuracy, and potential for radiation dose reduction using a novel iterative reconstruction technique-comparison with taditional filtered back projection. Eur Radiol..

[18] Gunn ML, Kohr JR (2010). State of the art: technologies for computed tomography dose reduction. Emerg Radiol..

[19] Fleischmann D, Boas FE (2011). Computed tomography - old ideas and new technology. Eur Radiol..

[20] Pontana F (2013). Reduced-dose low-voltage chest CT angiography with Sinogram-affirmed iterative reconstruction versus standard-dose filtered back projection. Radiology..

[21] Prakash P (2010). Reducing abdominal CT radiadion dose with adaptive statistical iterative reconstruction technique. Invest Radiol..

[22] Hara AK (2009). Iterative reconstruction technique for reducing body radiation dose at CT: feasibility study. AJR Am J Roentgenol..

[23] Remer EM (2014). Detection of urolithiasis: comparison of 100% tube exposure images reconstructed with filtered back projection and 50% tube exposure images reconstructed with sinogram-affirmed iterative reconstruction. Radiology..

[24] Iyama Y (2017). Feasibility of Iterative Model Reconstruction for Unenhanced Lumbar CT. Radiology..

[25] Yang CH (2016). Knowlege-based iterative model reconstruction technique in computed tomography of lumbar spine lowers radiation dose and improves tissue differentiation for patients with lower back pain. Eur J Radiol..

[26] Becce F (2013). Computed tomography of the cervical spine: comparison of image quality between a standard-dose and a low-dose protocol using filtered back-projection and iterative reconstruction. Skeletal Radiol..

[27] Geyer LL (2013). Evaluation of a dedicated MDCT protocol using iterative image reconstruction after cervical spine trauma. Clin Radiol..

[28] Omoumi P (2014). Low-dose multidetector computed tomography of the cervical spine: optimization of iterative reconstruction strength levels. Acta Radiol..

[29] Mei K (2017). Is multidetector CT-based bone mineral density and quantitative bone microstructure assessment at the spine still feasible using ultra-low tube current and sparse sampling?. Eur Radiol..

[30] Kopp FK (2016). Effect of Low-Dose MDCT and Iterative Reconstruction on Trabecular Bone Microstructure Assessment. Plos One.

[31] Mookiah MRK (2018). Multidetector Computed Tomography Imaging: Effect of Sparse Sampling and Iterative Reconstruction on Trabecular Bone Microstructure. J Comput AssistTomogr..

[32] Anitha D (2016). Effects of dose reduction on bone strength prediction using finite element analysis. Sci Rep..

[33] Geravaise A (2012). CT image quality improvement using Adaptive Iterative Dose Reduction with wide-voluem acquisition on 320-detector CT. Eur Radiol..

[34] Damilakis J, Adams JE, Guglielmi G, Link TM (2010). Radiation exposure in X-ray-based imaging techniques used in osteoporosis. Eur Radiol..

